# Beliefs and Perceptions About Parenteral Nutrition and Hydration by Advanced Cancer Patients

**DOI:** 10.1089/pmr.2022.0009

**Published:** 2022-08-08

**Authors:** Akiko Abe, Koji Amano, Tatsuya Morita, Tomofumi Miura, Naoharu Mori, Ryohei Tatara, Takaomi Kessoku, Yoshinobu Matsuda, Keita Tagami, Hiroyuki Otani, Masanori Mori, Tomohiko Taniyama, Nobuhisa Nakajima, Erika Nakanishi, Jun Kako, Daisuke Kiuchi, Hiroto Ishiki, Hiromichi Matsuoka, Eriko Satomi, Mitsunori Miyashita

**Affiliations:** ^1^ Department of Palliative Medicine, National Cancer Center Hospital, Tokyo, Japan.; ^2^ Department of Neuropsychiatry, Keio University School of Medicine, Tokyo, Japan.; ^3^ Department of Palliative and Supportive Medicine, Graduate School of Medicine, Aichi Medical University, Nagakute City, Japan.; ^4^ Palliative and Supportive Care Division, Seirei Mikatahara General Hospital, Hamamatsu City, Shizuoka, Japan.; ^5^ Department of Palliative Medicine, National Cancer Center Hospital East, Kashiwa City, Japan.; ^6^ Department of Palliative Medicine, Osaka City General Hospital, Osaka City, Japan.; ^7^ Department of Palliative Medicine, Yokohama City University Hospital, Yokohama City, Japan.; ^8^ Department of Gastroenterology and Hepatology, Yokohama City University Graduate School of Medicine, Yokohama City, Japan.; ^9^ Department of Psychosomatic Internal Medicine, National Hospital Organization Kinki-Chuo Chest Medical Center, Sakai City, Japan.; ^10^ Department of Palliative Medicine, Tohoku University Graduate School of Medicine, Sendai City, Japan.; ^11^ Department of Palliative Care Team, and Palliative and Supportive Care, St. Mary's Hospital, Kurume City, Japan.; ^12^ Department of Clinical Oncology and Palliative Medicine, Mitsubishi Kyoto Hospital, Kyoto City, Japan.; ^13^ Division of Community Medicine and International Medicine, University of the Ryukyus Hospital, Nakagami-Gun, Japan.; ^14^ Department of Palliative Nursing, Health Sciences, Tohoku University Graduate School of Medicine, Sendai City, Japan.; ^15^ Graduate School of Public Health, St. Luke's International University, OMURA Susumu and Mieko Memorial St. Luke's Center for Clinical Academia, Tokyo, Japan.; ^16^ College of Nursing Art and Science, University of Hyogo, Akashi City, Japan.; ^17^ Department of Psycho-Oncology, National Cancer Center Hospital, Tokyo, Japan.

**Keywords:** advanced cancer, cachexia, nutritional support, palliative care, parenteral hydration, parenteral nutrition

## Abstract

**Background:**

The beliefs and perceptions of parenteral nutrition and hydration (PNH) by advanced cancer patients have not been elucidated.

**Objectives:**

To clarify their beliefs and perceptions and to explore the relationships between their beliefs and perceptions and cachexia stages.

**Design/setting/subjects:**

A questionnaire survey of advanced cancer patients receiving palliative care across Japan.

**Measurements:**

We asked patients to answer 15 items regarding their beliefs and perceptions of PNH. Frequencies were calculated for the patient characteristics and survey parameters. Comparisons were performed using the Mann–Whitney U test. We conducted a factor analysis and a multiple logistic regression analysis to identify the independent factors affecting cancer cachexia stages.

**Results:**

Among 495 patients, 378 responded. Due to missing data, 357 remained in the frequency distribution analysis, and 344 were classified into the noncachexia group (*n* = 174) and cachexia group (*n* = 170). Approximately 60% thought that PNH were beneficial. Approximately 70% considered PNH a standard medical practice. Approximately 70% did not feel that they received a sufficient explanation. There were no significant differences in any items between the two groups. We extracted four conceptual groups. The concept of “Belief that PNH are harmful” was identified as an independent factor [odds ratio 2.57 (95% confidence intervals 1.10–6.01), *p* = 0.030].

**Conclusion:**

More than half of the patients thought that PNH were beneficial and standard medical practices with or without cancer cachexia. The negative perception of PNH decreased in patients with cancer cachexia.

## Introduction

The 2017 European Society for Clinical Nutrition and Metabolism guidelines strongly recommends the following: (1) screening/monitoring for nutrition risk in all patients with cancers of advanced stage, with in-depth nutritional assessment for patients who screen positive and (2) providing nutritional counseling and oral nutritional supplements as the first-line approach, with escalation to parenteral nutrition and hydration (PNH) according to specified criteria.^1^

Evidence-based clinical practice guidelines for management of cancer cachexia and nutritional care edited by the American Society of Clinical Oncology and European Society for Medical Oncology suggest that the provision of parenteral nutrition (PN) to manage cachexia in patients with advanced cancer is not recommended, that PN should not be initiated in the last weeks of life, and that discontinuation of previously initiated PN near the end of life is appropriate.^2–4^ However, the evidence quality is low in these clinical guidelines.^1–4^

While a more recent randomized controlled trial demonstrated that PN did not improve the quality of life or survival of cancer patients with functional gastrointestinal tract and a median survival of 2.5 months,^5^ a prospective multicenter cohort study conducted in palliative care units implied the beneficial effects of PNH on survival and quality of dying among cancer patients with a mean survival of five weeks.^6–8^ Thus, there is currently no consistency in findings and a paucity of evidence on the beneficial effects of PNH in advanced cancer, which has led to diversity in daily clinical practice.^1–4^

In decisions regarding the use of PNH, beliefs and perceptions about PNH by patients and families cannot be ignored. Previous studies reported that the majority of cancer patients and families wanted nutritional support to be initiated when patients became unable to intake a sufficient amount of food orally and that patients with cancer cachexia expressed a greater need for nutritional support.^9–13^ A large number of patients and families wished to receive PNH rather than tube feeding.^11,13^ An unmet need for PNH may be connected with eating-related distress experienced by patients and families.^14^ However, it is unclear whether patients and families were able to distinguish between PN and parenteral hydration (PH), which may also lead to diversity in daily clinical practice.^11,13^

To the best of our knowledge, the beliefs and perceptions of PNH, PN, and PH by patients with advanced cancer and changes of the beliefs and perceptions in disease trajectory have not been elucidated. Furthermore, on the basis of the above, we hypothesized that patients with cancer cachexia had a stronger preference for receiving PNH. Therefore, we conducted a preplanned secondary analysis of a questionnaire survey of patients with advanced cancer in palliative care settings to clarify their beliefs and perceptions about PNH, clearly distinguishing between PN and PH, and to explore the relationships between their beliefs and perceptions and cachexia stages based on the criteria from the international consensus.^15^

## Methods

This study was performed as part of a multicenter self-report questionnaire survey conducted at 11 hospitals across Japan between July 2020 and July 2021.

Consecutive eligible patients were enrolled. The inclusion criteria were as follows: (1) patients newly referred to palliative care, (2) patients ≥20 years old, (3) patients with locally advanced or metastatic cancer (hematological neoplasms were included), (4) patients with awareness of the diagnosis of malignancy, and (5) patients with the ability to reply to a self-reported questionnaire. The exclusion criteria were as follows: (1) patients forbidden to eat by the physician, and (2) psychological issues recognized in an interview with the physician. If subjects did not want to participate, we requested them to return the questionnaire with “no participation” indicated. The completion and return of the questionnaire were regarded as consent to participate in this study. Ethical approval for this study was granted by the Institutional Review Board of each hospital.

### Questionnaires

The questionnaire for this study was developed by the authors based on a previous survey of bereaved families.^13^ The face validity of the questionnaire was confirmed by a pilot test with five medical personnel, five physicians, and three nurses.

We asked about patient characteristics. We also asked patients to report on dietary intakes with the ingesta-Verbal/Visual Analogue Scale, using the 10-point analogue scales (high scores indicate better dietary intakes).^16^

We requested patients to report anthropometric measurements to calculate body mass index (BMI) and % weight loss (WL) in six months.

We finally asked patients to answer 15 items regarding their beliefs and perceptions about PNH using the following seven-point Likert scale: (1) absolutely agree, (2) agree, (3) somewhat agree, (4) not either, (5) somewhat disagree, (6) disagree, and (7) absolutely disagree. In the questionnaire, we explained PN/PH as “supplying nutrition/hydration through an intravenous drip” in easy Japanese.

### Statistical analyses

Patient characteristics were presented as *n* (%) or medians (interquartile ranges) where appropriate.

BMI was calculated by dividing current body weight (kg) by height (m).^2^ %WL was calculated as follows: (current body weight [kg] − previous body weight [kg])/previous body weight (kg) × 100. Cachexia was %WL in 6 months ≥5% or BMI <20 kg/m^2^ + %WL in 6 months ≥2%. Patients above or below these cutoff values were grouped as follows: the noncachexia group and cachexia group.^15^

The proportions of patients with “absolutely agree,” “agree,” or “somewhat agree” were calculated regarding the 15 items about their beliefs and perceptions of PNH. Comparisons of the scores for the 15 items between the noncachexia and cachexia groups were performed using the Mann–Whitney U test.

We conducted an exploratory factor analysis using the principle method with a promax rotation. We calculated Cronbach's alpha coefficients to assess the internal consistency of a set of items in each of the conceptual groups extracted.

A multiple logistic regression analysis was performed to identify the independent factors affecting cancer cachexia stages using patient characteristics and the mean scores for items in each concept of patients' beliefs and perceptions, which were dichotomized with <4 (absolutely agree, agree, and somewhat agree) or ≥4 (not either, somewhat disagree, disagree, and absolutely disagree). A multivariate model was adjusted for sex, age, the primary cancer site, and Eastern Cooperative Oncology Group performance status (ECOG PS).

All results were considered to be significant when the *p*-value was <0.05. All analyses were performed using SPSS software version 27.0.

## Results

A total of 495 patients were asked to take part in this survey, and 378 responded (76.4%). None of these patients indicated “no participation.” Twenty-one patients were excluded due to missing data on beliefs and perceptions about PNH, and thus, 357 remained in the frequency distribution analysis and exploratory factor analysis. Following the exclusion of 13 patients due to missing data on the classification of cachexia stages, 344 were classified into the noncachexia group (*n* = 174) and cachexia group (*n* = 170).

### Patient characteristics

Males accounted for 50.8% of patients and the median age was 63.0 years. The lungs were the most common primary cancer site. The proportions of ECOG PS 0 to 1, 2, and 3 to 4 were 45.7%, 19.2%, and 27.5%, respectively. The proportion of outpatient service was 67.9% and that of chemotherapy was 60.4% (Table 1).

**Table 1.** Patient Characteristics (*n* = 378)

Sex	
Male	192 (50.8)
Female	181 (47.9)
Age in years	63.0 (53.0–72.0)
Age	
<65	201 (50.8)
65–74	111 (28.0)
≥75	61 (15.4)
Primary cancer site	
Upper and lower gastrointestinal tract	52 (13.1)
Liver, biliary system, and pancreas	62 (15.7)
Lungs	87 (22.0)
Others	167 (42.2)
ECOG performance status	
0–1	181 (45.7)
2	76 (19.2)
3–4	109 (27.5)
Setting of care	
Outpatient service	269 (67.9)
Hospital palliative care team	89 (22.5)
Palliative care unit	13 (3.3)
Treatment status	
Prechemotherapy	24 (6.1)
Chemotherapy	239 (60.4)
Never treated/previous treatment	103 (26.0)
Dietary intake	6.0 (4.0–8.0)
Body mass index (kg/m^2^)	20.8 (18.5–23.5)
Weight loss in one month, yes	158 (48.8)
Cachexia, yes	170 (45.0)
Symptomatic fluid retention, yes	80 (21.2)

Values represent *n* (%) or medians (interquartile ranges) where appropriate. The sums of some percentages do not add up to 100% due to missing values. Dietary intakes were measured with the ingesta-Verbal/Visual Analogue Scale using 10-point analogue scales. Cachexia was based on criteria from the international consensus.

ECOG, Eastern Cooperative Oncology Group.

### Prevalence of beliefs and perceptions about PNH

In items regarding preferences for PN and PH (Q1–6), 56.9% and 69.2% of patients thought that PN and PH were substitutes for oral nutrition and hydration, respectively. A total of 66.9% and 70.8% of patients preferred to receive PN and PH, respectively. A total of 77.5% and 81.4% of patients needed PN and PH for their families, respectively. The preference for PH was consistently higher than that for PN. In items regarding perceptions of PNH (Q7–12), 68.4% of patients thought that PNH need to be a standard medical practice.

**FIG. 1.**
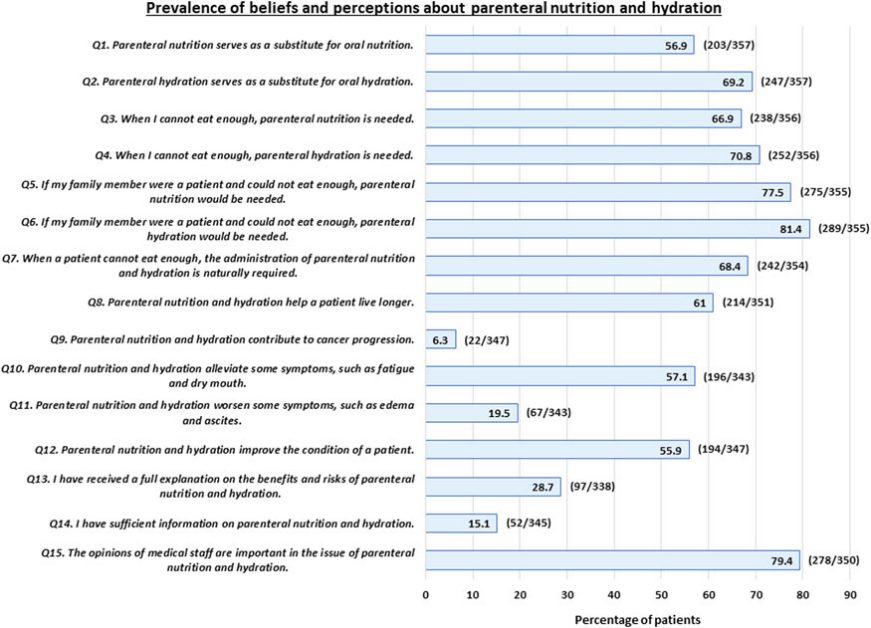
Prevalence of beliefs and perceptions about parenteral nutrition and hydration. The percentages represent the number of patients giving “absolutely agree, agree, or somewhat agree.”

A total of 55.9% to 61.0% of patients thought that PNH were beneficial, while they rarely considered PNH to be harmful (6.3–19.5%). In items regarding the explanation of and information on PNH (Q13–15), patients rarely thought that they received a full explanation/sufficient information (15.1–28.7%), and 79.4% of patients depended on medical staff to make a decision on PNH (Fig. 1).

### Comparison between the noncachexia group and cachexia group

There were no significant differences in any items between the two groups (Table 2).

**Table 2.** Comparison of Beliefs and Perceptions About Parenteral Nutrition and Hydration between the Noncachexia Group and Cachexia Group (*n* = 344)

Items	Noncachexia	Cachexia	***p***
Q1. PN serves as a substitute for oral nutrition.	3.5 ± 1.7, 3.0	3.5 ± 1.8, 3.0	0.75
Q2. PH serves as a substitute for oral hydration.	3.0 ± 1.5, 3.0	3.0 ± 1.5, 3.0	0.66
Q3. When I cannot eat enough, PN is needed.	3.2 ± 1.6. 3.0	3.0 ± 1.6, 3.0	0.31
Q4. When I cannot eat enough, PH is needed.	3.1 ± 1.6, 3.0	2.8 ± 1.5, 2.0	0.10
Q5. If my family member were a patient and could not eat enough, PN would be needed.	2.7 ± 1.4, 2.0	2.6 ± 1.5, 2.0	0.32
Q6. If my family member were a patient and could not eat enough, PH would be needed.	2.5 ± 1.3, 2.0	2.4 ± 1.3, 2.0	0.10
Q7. When a patient cannot eat enough, the administration of PNH is naturally required.	2.8 ± 1.3, 3.0	3.0 ± 1.5, 3.0	0.44
Q8. PNH help a patient live longer.	3.1 ± 1.3, 3.0	3.3 ± 1.5, 3.0	0.56
Q9. PNH contribute to cancer progression.	5.1 ± 1.3, 5.0	5.2 ± 1.3, 5.0	0.32
Q10. PNH alleviate some symptoms, such as fatigue and dry mouth.	3.5 ± 1.4, 3.0	3.3 ± 1.3, 3.0	0.14
Q11. PNH worsen some symptoms, such as edema and ascites.	4.3 ± 1.2, 4.0	4.5 ± 1.3, 4.0	0.10
Q12. PNH improve the condition of a patient.	3.4 ± 1.2, 3.0	3.3 ± 1.2, 3.0	0.51
Q13. I have received a full explanation on the benefits and risks of PNH.	4.1 ± 1.5, 4.0	4.1 ± 1.5, 4.0	0.89
Q14. I have sufficient information on PNH.	4.9 ± 1.6, 5.0	4.8 ± 1.5, 5.0	0.30
Q15. The opinions of medical staff are important in the issue of PNH.	2.6 ± 1.4, 2.0	2.5 ± 1.4, 2.0	0.50

Values represent means ± standard deviations and medians.

PH, parenteral hydration; PN, parenteral nutrition; PNH, parenteral nutrition and hydration.

### Exploratory factor analysis and internal consistency of a set of items

We extracted four conceptual groups as follows: “Belief that PNH are a standard medical practice I want,” “Belief that PNH are beneficial,” “Perception that knowledge about PNH is enough,” and “Belief that PNH are harmful.” Cronbach's alpha coefficients were 0.87, 0.73, 0.71, and 0.48, respectively (Table 3).

**Table 3.** Factor Validity of Beliefs and Perceptions About Parenteral Nutrition and Hydration: Four Core Domains (*n* = 357)

	Standardized regression coefficients				Communality
	F1	F2	F3	F4	
F1: Belief that PNH are a standard medical practice I want (mean = 2.79, SD = 1.48, Cronbach's α = 0.87)					
Q5. If my family member were a patient and could not eat enough, PN would be needed.	**0.993**	−0.209	0.068	−0.035	0.817
Q6. If my family member were a patient and could not eat enough, PH would be needed.	**0.918**	−0.161	0.036	−0.054	0.715
Q3. When I cannot eat enough, PN is needed.	**0.712**	0.183	−0.034	0.031	0.678
Q4. When I cannot eat enough, PH is needed.	**0.650**	0.238	−0.049	−0.013	0.640
Q7. When a patient cannot eat enough, the administration of PNH is naturally required.	**0.469**	0.125	−0.021	0.017	0.298
F2: Belief that PNH are beneficial (mean = 3.15, SD = 1.47, Cronbach's α = 0.73)					
Q12. PNH improve the condition of a patient.	−0.101	**0.872**	0.076	−0.076	0.695
Q10. PNH alleviate some symptoms, such as fatigue and dry mouth.	−0.071	**0.658**	−0.005	0.029	0.390
Q2. PH serves as a substitute for oral hydration.	0.347	**0.456**	−0.077	0.000	0.491
Q8. PNH help a patient live longer.	0.208	**0.350**	−0.004	0.123	0.265
Q1. PN serves as a substitute for oral nutrition.	0.320	**0.343**	−0.018	0.105	0.352
Q15. The opinions of medical staff are important in the issue of PNH.	0.066	**0.255**	0.138	−0.116	0.122
F3: Perception that knowledge about PNH is enough (mean = 4.47, SD = 1.57, Cronbach's α = 0.71)					
Q14. I have sufficient information on PNH.	−0.021	0.054	**0.735**	0.013	0.552
Q13. I have received a full explanation on the benefits and risks of PNH.	0.046	0.040	**0.709**	0.019	0.523
F4: Belief that PNH are harmful (mean = 4.76, SD = 1.35, Cronbach's α = 0.48)					
Q9. PNH contribute to cancer progression.	−0.011	−0.091	0.042	**0.776**	0.612
Q11. PNH worsen some symptoms, such as edema and ascites.	−0.026	0.069	−0.012	**0.459**	0.216
Cumulative proportion, 49.1%					

Boldfaced numbers indicate attributes belonging to each domain. F#, Factors 1 to 4; Cronbach's α, Cronbach's alpha coefficients.

SD, standard deviation.

### Multiple logistic regression analysis

The results of a multiple logistic regression analysis are shown in Table 4. The concept of “Belief that PNH are harmful” was identified as an independent factor [odds ratio 2.57 (95% confidence intervals 1.10–6.01), *p* = 0.030].

**Table 4.** Estimated Crude and Adjusted Odds Ratios for a Logistic Regression Model Assessing the Effect of Beliefs and Perceptions About Parenteral Nutrition and Hydration on Cachexia Stages (*n* = 344)

	Crude OR (95% CI)	***p***	Adjusted OR (95% CI)	***p***
Sex				
Male	1.00 (reference)		1.00 (reference)	
Female	0.81 (0.53–1.24)	0.32	1.02 (0.62–1.68)	0.94
Age				
<65	1.00 (reference)		1.00 (reference)	
65–74	1.99 (1.21–3.27)	0.007	2.08 (1.15–3.76)	0.015
≥75	0.83 (0.45–1.53)	0.55	1.10 (0.53–2.27)	0.80
Primary cancer site				
Upper and lower gastrointestinal tract	1.00 (reference)		1.00 (reference)	
Liver, biliary system, and pancreas	1.28 (0.60–2.73)	0.52	1.16 (0.50–2.73)	0.73
Lungs	1.39 (0.69–2.83)	0.36	1.11 (0.50–2.47)	0.79
Others	0.84 (0.44–1.60)	0.60	0.72 (0.35–1.49)	0.37
ECOG performance status				
0–1	1.00 (reference)		1.00 (reference)	
2	1.23 (0.71–2.13)	0.47	1.15 (0.61–2.16)	0.66
3–4	2.03 (1.23–3.36)	0.006	1.96 (1.09–3.54)	0.025
Factor 1				
Absolutely agree, agree, and somewhat agree (mean scores <4)	1.00 (reference)		1.00 (reference)	
Not either, somewhat disagree, disagree, and absolutely disagree (mean scores ≥4)	0.61 (0.34–1.10)	0.10	0.79 (0.39–1.63)	0.53
Factor 2				
Absolutely agree, agree, and somewhat agree (mean scores <4)	1.00 (reference)		1.00 (reference)	
Not either, somewhat disagree, disagree, and absolutely disagree (mean scores ≥4)	0.72 (0.41–1.27)	0.26	0.84 (0.41–1.69)	0.62
Factor 3				
Absolutely agree, agree, and somewhat agree (mean scores <4)	1.00 (reference)		1.00 (reference)	
Not either, somewhat disagree, disagree, and absolutely disagree (mean scores ≥4)	0.79 (0.48–1.31)	0.36	0.74 (0.42–1.31)	0.30
Factor 4				
Absolutely agree, agree, and somewhat agree (mean scores <4)	1.00 (reference)		1.00 (reference)	
Not either, somewhat disagree, disagree, and absolutely disagree (mean scores ≥4)	1.64 (0.79–3.43)	0.19	2.57 (1.10–6.01)	0.030

Thirty-four subjects were excluded due to missing data: beliefs and perceptions about PNH (*n* = 21) and cachexia stages (*n* = 13). A multivariate model adjusted for sex, age, the primary cancer site, and ECOG performance status.

OR, odds ratio; CI, confidence interval.

## Discussion

This is the first survey of patients with advanced cancer to clarify their beliefs and perceptions about PN, PH, and PNH using the division into cachexia and noncachexia groups.

Approximately 60% of patients thought that PNH were beneficial. Approximately 70% thought that PNH were a standard medical practice. However, more than 70% to 80% of patients did not feel that they received sufficient explanation/information or that they had adequate knowledge. Therefore, a large number of patients were dependent on their primary physicians.

The previous survey of the bereaved families of cancer patients reported that 60% to 80% believed that PNH were beneficial and that 80% to 90% expressed a need for PNH when the patient was unable to intake a sufficient amount of food. More than 70% had insufficient information on PNH and more than 50% did not receive a full explanation about PNH. Families were also likely to depend on medical staff when they had to make a decision for their loved one.^13^

Patients and families had similar preferences regarding PNH; however, families were reluctant to withhold PNH for their loved one even if the patient confidently decided to forgo PNH.^17^ Furthermore, the majority of patients considered their families' opinions to be crucial for making decisions regarding PNH.^17^ Differences in the perception of this matter may generate conflict, leading to eating-related distress experienced by patients and families.^14^

Every patient requires individualized nutritional support, which needs to be considered along with the intention of patients and families. However, medical staff need to address the imbalance between the hope of patients and families and reality with the provision of correct information and education when PNH appear to be disproportionate care.^18,19^

Regarding the relationships between patients' beliefs and perceptions about PNH and cachexia stages, no significant differences were observed between the noncachexia and cachexia groups. The results obtained also identified the concept of “Belief that PNH are harmful” as an independent factor. However, the value of Cronbach's alpha coefficient of “Belief that PNH are harmful” was low. This may be because only two items were categorized into this factor.

Several reasons need to be considered as follows. Patients are likely to think that PNH are a standard medical practice, that PNH are beneficial, and that knowledge about PNH is not sufficient with or without cancer cachexia. In contrast, they rarely feel that PNH are harmful with or without cancer cachexia; however, the negative perception of PNH may decrease if cachexia-related symptoms become more apparent. The previous surveys, which demonstrated that patients with cachexia had more severe cachexia-related symptoms and greater eating-related distress than those without cachexia^11^ and that patients with cachexia expressed a greater need for nutritional support,^10^ appear to support this result. Further studies are warranted.

This study has several limitations. As many patients who were in good performance status and received chemotherapy were included, the situation may be different from previous studies enrolling patients in the end-of-life periods.^20–23^ The questionnaire, which has not been validated, may lead to confirmation bias. Settings where PNH are provided were not clearly described in the questionnaire. However, visiting physicians also administer PNH, as well as tube feeding, to patients living in their homes in Japan. Since this study used a cross-sectional analysis, survival data and information on PNH treatments were not obtained.

## Conclusion

More than half of the patients with advanced cancer thought that PNH were beneficial and standard medical practices despite insufficient information, and they had a moderate preference for receiving PNH with or without cachexia. The negative perception of PNH decreased in patients with cachexia.

## Abbreviations Used

BMI: body mass index
CI: confidence interval
ECOG: Eastern Cooperative Oncology Group
ECOG PS: Eastern Cooperative Oncology Group performance status
OR: odds ratio
PH: parenteral hydration
PN: parenteral nutrition
PNH: parenteral nutrition and hydration
SD: standard deviation
WL: weight loss
